# The Effect of Inhalation of Aromatherapy Blend containing Lavender Essential Oil on Cesarean Postoperative Pain

**DOI:** 10.5812/aapm.9570

**Published:** 2013-07-01

**Authors:** Alireza Olapour, Kaveh Behaeen, Reza Akhondzadeh, Farhad Soltani, Forough al Sadat Razavi, Reza Bekhradi

**Affiliations:** 1 Department of Anesthesiology, Golestan Hospital, Ahvaz Jundishapur University of Medical Sciences, Ahvaz, Iran; 2 Barij Essence Research and Development Centre, Kashan, Iran

**Keywords:** Pregnant Women, Aromatherapy, Lavandula, Cesarean Section, Pain

## Abstract

**Background:**

Pain is a major problem in patients after cesarean and medication such as aromatherapy which is a complementary therapy, in which the essences of the plants oils are used to reduce such undesirable conditions.

**Objectives:**

In this study, the effect of aromatherapy using Lavender (Lavandula) essential oil on cesarean postoperative pain was assessed.

**Materials and Methods:**

In a triple blind, randomized placebo-controlled trial study, 60 pregnant women who were admitted to a general hospital for cesarean section, were divided randomly into two groups. After cesarean, the Lavender group inhaled about 3 drops of 10% Lavender oil essence and the placebo group inhaled 3 drops of placebo after the start of postoperative pain, four, eight and 12 hours later, for 5 minutes from the 10 cm distance. Patient's pain was measured by the VAS (Visual Analog Scale) score before and after each intervention, and vital sign, complications and level of satisfaction of every patient were recorded before and after aromatherapy.

**Results:**

There was no statistically significant difference between groups in age, height, weight, and time to the first analgesic requirement. Patients in the Lavender group had less postoperative pain in four (P = 0.008), eight (P = 0.024) and 12 (P = 0.011) hours after first medication than the placebo group. The decreased heart rate and patients' level of satisfaction with analgesia were significantly higher in the Lavender group (P = 0.001). In the placebo group, the use of diclofenac suppositories for complete analgesia was also significantly higher than the Lavender group (P = 0.008).

**Conclusions:**

The inhaled Lavender essence may be used as a part of the multidisciplinary treatment of pain after cesarean section, but it is not recommended as the sole pain management.

## 1. Background

Pain is amongst the most common problems after surgery (1). Pain is an unpleasant sensory and emotional experience, which is associated with the real or probably damage of tissue. Unrelieved postoperative pain in addition to creating fears in the surgical patients, it makes adverse psychological impact on them (2, 3). The uncontrolled postoperative pain will make a lot of acute and chronic effects, including systemic mediators, hypercoagulability, postoperative immunosuppression, and delayed wound healing (4). So, one of the main aims of anesthesia is to reduce postoperative pain. However, many drugs that are used for this purpose, especially opioids and NSAIDs (None Steroidal Anti Inflammatory Drugs), have side effects such as respiratory distress, nausea, itching, and gastrointestinal bleeding (5). Recent studies have indicated interest in using complementary therapies such as heat and cold therapy, hypnotism, music therapy and aromatherapy. Aromatherapy is used for the relief of pain, anxiety, depression, insomnia and fatigue, using the existing oils in different parts of the plant such as Lavandula angustifolia (6-11). Lavandula is a flowering plant from the Lamiaceae family, native to the western Mediterranean region. The lipophilic monoterpenes at the plant are reacted to the cell membranes, and cause changes in the activity of ion channels, carriers and nervous receptors. Such property can explain the soothing and anti-bacterial effects of Lavender oil (12).

## 2. Objectives

Cesarean surgery is common, especially in our country, and the patient's postoperative pain is a serious problem. Furthermore, the extent of conventional method for the pain relief is a prescription of opioids and NSAIDs, which are also associated with certain complications, and needs additional methods of the pain control, consequently, we decided to assess the effect of the inhalation aromatherapy using Lavender oil essence on postoperative pain.

## 3. Materials and Methods 

In a triple blind, randomized placebo-controlled trial, which was performed for the first time in Ahvaz, a city located in the south western part of Iran, after approving by Ahvaz Jundishapur University of Medical Sciences (AJUMS) Ethical Committee, 60 pregnant women, who were admitted to a general hospital for cesarean section, were divided randomly into two groups. Subjects with pregnancy, ASA class I and II, absence of hypertension, coagulation disorders, migraines and chronic headaches, no history of allergies to medicinal plants, no history of anosmia were included. Subjects with respiratory problems during surgery, nausea, vomiting, sensitivity and dissatisfaction after the first dose of aromatherapy using Lavender oil essence were excluded. The severity of pain was documented based on the Visual Analog Scale (VAS).The VAS is a standard tool like a 10 cm ruler including 10 numbers begin from 0 (no pain) and end to 10 (most severe pain). Different states of a human face in response to pain severity have been plotted on the other side of ruler. The patients were asked to choose one of them according to their pain severity. The number shown on the back of the ruler was considered as pain score. The Lavenders (Lavandula angustifolia) are a genus of several species of flowering plants in the mint family, Lamiaceae. In this study, Lavender essence 10% was provided by the Barij Essence Pharmaceudical Company (Kashan, Iran). Placebo was a base of aromatherapy blend without Lavender essence which was provided by the Barij Essence Pharmaceudical Company too. In the beginning, possible side effects of drugs used in the study, were explained to the patients, and after obtaining informed consent, patients were entered into the operating room. Patients were performed monitoring by ECG (Electrocardiography), monitoring for heart rate recording, NIBP (None Invasive Blood Pressure) and Pulseoximetry. After embedding the peripheral intravenous cannula, 500 cc of Ringer's crystalloid fluid was infused for patients. Afterwards, regional block with spinal anesthesia was performed with 60 mg of Lidocaine 5%, and then the patients underwent cesarean section. Opioid or benzodiazepine was not used after cesarean section in operating room as sedation. Pain score were measured using the VAS score for all patients. After the onset of postoperative pain (if VAS > 3), four, eight and 12 hours after that, the inhalation aromatherapy was performed using Lavender essence. In one group, three drops of aromatherapy blend containing Lavender essence 10% (provided by The Barij Essence Pharmaceutical Compnay) were poured on cotton in cast containers, and the patient was asked to inhale it for 5 minutes from a distance of 10 cm; and pain score was measured using the VAS again, and if the VAS was greater than three, analgesic was given in accordance with the hospital routine protocol (the first time, intra muscular injection of Diclofenac sodume 75 mg and next times, Diclofenac suppositories 100 mg). Using the same procedure, aromatherapy was performed in the other group by three drops of placebo (a base of aromatherapy blend without Lavender essence) where its smell and appearance were similar to the Lavender oil essence. Heart rate, blood pressure, nausea, vomiting, dizziness, and patient's satisfaction were recorded before and after the aromatherapy based on the questionnaire. During the research, the project executive and the patients were not aware of the type of drug and placebo, and after the study and data analysis, the Lavender and the placebo were disclosed by the Barij Essence Pharmaceudical Company. All data were analyzed using the SPSS for Windows (version 19.0). Independent T-test was used compare the mean pain in two groups; the Paired t- test was used to compare the pain before and after intervention. The significance level was set to P ≤ 0.05.

## 4. Results

**Figure 1.**
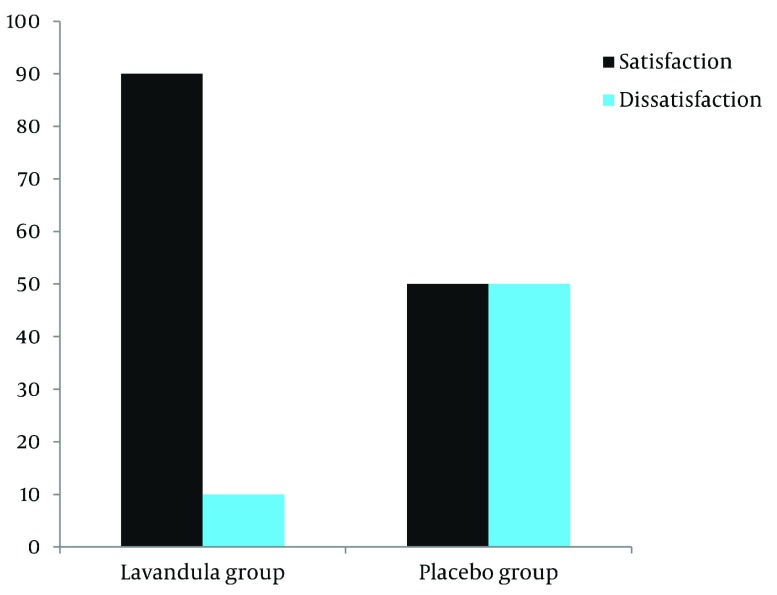
The Satisfaction From Using Drug in Two Groups

**Figure 2.**
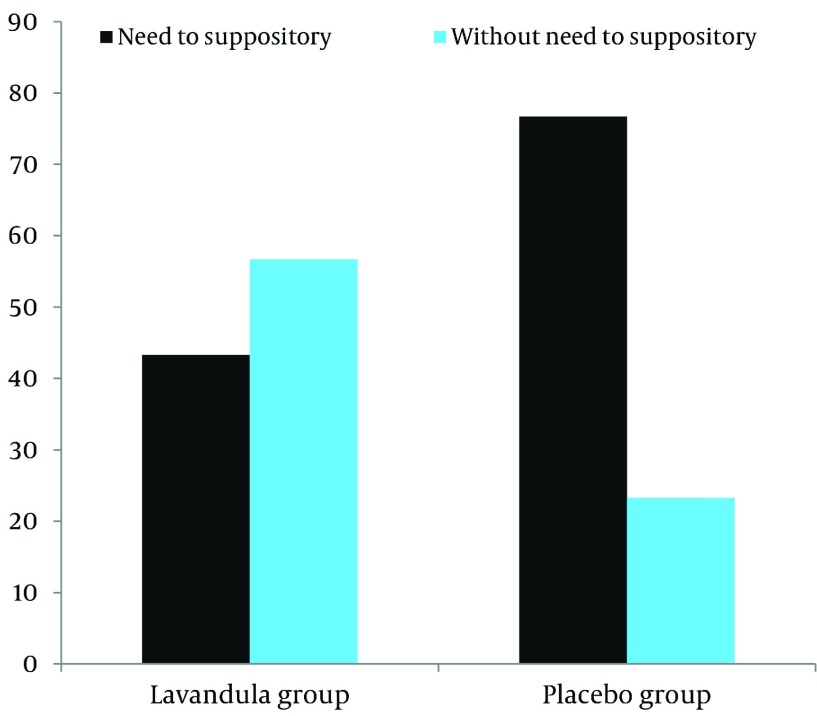
Frequency of Patients need to Take Diclofenac Suppositories to Complete Pre-Operative Analgesia

In this study, all patients were ASA class I. At the time of the onset of the pain after cesarean section, aromatherapy with Lavender essence was performed. Average age, height, weight in the two groups showed no significant difference, and times of need to the first analgesic from cesarean section were similar in the two groups (Table 1). After using the drug comparing it with before, there was more decrease in the VAS score in the Lavender group than the placebo group, these values were significant in four, eight and 12 hours after the first intervention (Table 2). In the Lavender group, the level of satisfaction from the drug was 90 %, while in the placebo group, a 50% satisfaction was reported (P = 0.001) (Figure 1). In the Lavender group, using Diclofenac suppository for completing analgesia was 43.3%, and in the placebo group was 76.7% (P = 0.008) (Figure 2).

After using the drug comparing it with before, heart rate showed a greater reduction in the Lavender group compared with the placebo group, which has been shown in the table (Table 3). However, no difference was observed in terms of the blood pressure between the two groups. In terms of the complication incidence, only one patient in the placebo group had nausea, and none of the patients in both groups had vomiting and dizziness.

**Table 1.** Baseline Charactristics of Patients

	Lavender group, Mean ± SD	Placebo group, Mean ± SD	P value
Age, y	27.83 ± 5.65	25.57 ± 4.11	0.96
Height, cm	159.57 ± 4.15	158.7 ± 4.41	0.78
Weight, kg	79.57 ± 8.99	76.07 ± 9.35	0.78
Time of first request of analgesia after Spinal anesthesia	98.83 ± 9.16	98.33 ± 9.67	0.72

**Table 2.** Comparison of the VAS in Two Groups

	Lavender group, Mean ± SD	Placebo group, Mean ± SD	P value
Pain reduction after medication for the first time	0.23 ± 0.43	0.27 ± 0.64	0.353
Pain reduction 4 hours after medication for the first time	1.37 ± 0.89	0.5 ± 0.57	0.008
Pain reduction 8 hours after medication for the first time	1.63 ± 0.89	0.4 ± 0.49	0.024
Pain reduction12hours after medication for the first time	1.40 ± 0.62	0.2 ± 0.48	0.011

**Table 3.** Comparison of Heart Rate in Two Groups

	Lavender group, Mean ± SD	Placebo group Mean ± SD	P value
Pain reduction after medication for the first time	4.83 ± 5.96	0.63 ± 2.45	< 0.001
Pain reduction 4 hours after medication for the first time	3.30 ± 4.60	0.77 ± 4.24	0.439
Pain reduction 8 hours after medication for the first time	4.30 ± 5.62	0 ± 3.16	0.016
Pain reduction12hours after medication for the first time	2.2 ± 2.82	0.57 ± 2.23	0.798

## 5. Discussion

This study investigated the effect of inhaled Lavender essence on the pain relief after cesarean. The results showed that pain after surgery in four, eight and 12 hours after the onset of symptoms following inhalation of Lavender essence have had a significant decrease compared with the placebo group. The pain control after cesarean delivery is a great challenge for the anesthesiologists and gynecologists, because the spread use of drugs can cause side effects such as nausea, vomiting and excessive sedation; and it can cause a delay in getting out of bed and discharge from the hospital. In addition, the drugs excrete in breast milk and can cause sedation in baby as well (13). The use of non-opioid analgesics and alternative treatment alone has failed to establish an effective and satisfactory analgesia for patients. It seems necessary to use multiple methods of analgesia (multi-modal) for the pain relief after Cesarean section and to have more research in this area (4). The effects of various forms of Lavender essence on postoperative pain control have been investigated in the previous studies. In a study performed by Sobhani and Colleagues in 2004 in Rasht city on pain relief after cesarean section, in each three stages of the intervention (in which its first stage was performed six hours after the onset of the pain) there was a significant reduction in pain after inhaling Lavender essence. These results are similar to our study except that: 1) in the mentioned study, intervention was not immediately performed after the onset of the pain and 2) we used Lavender essence 10% (14). In a study on women undergoing elective cesarean section performed in Tabriz city, an important reduction was seen in pain at half, eight and 16 hours after intervention in the group using inhaled Lavender, while in our study, there was no significant difference between the two groups at the first intervention at the onset of the pain unlike for the four, eight and 12 hours. Also in our study, the Lavender essence 10% was used (6). In a study performed by Sheikhan and Colleagues on episiotomy pain using Lavender bath extract, pain in the Lavender group at four hours and also five days after delivery was significantly reduced (9). Moreover, in a similar study conducted by Ailsa also using Lavender oil bath for 10 days after vaginal delivery, the slight reduction of pain, though significant, was seen in the Lavender group compared to the control group (15). Furthermore, in Khadivzadeh's study in which Lavender cream has been used to relieve episiotomy pain, the pain on days three, five and 10 after birth was significantly reduced compared to the control group, but this difference was not significant in the first 24 hours of the delivery (16). We found that Diclofenac suppository dosage as a supplemental analgesic drug in the Lavender group was significantly lower than the placebo group. These findings are similar to a study by Jung TK on patients who underwent laparoscopic gastric banding surgery. But in our study the Lavender essence 10% was used. In that study, despite the decrease of pain, the score was not significantly different in the two groups, but morphine consumption was decreased significantly in the Lavender group (17). In the present study, patient's satisfaction with supplemental analgesia techniques was considered, and it showed that satisfaction with treatment in the Lavender group was significantly higher than the placebo group. This satisfaction has also been reported in two previous studies, which were conducted on obese patients undergoing laparoscopic adjustable gastric banding and patients undergoing breast biopsy surgery with the difference that we used Lavender essence 10% (17, 18). Also, in a study conducted by Mohammadkhani on postpartum perineal pain, patients receiving Lavender were more satisfied than controls (19). Due to the lack of reported side effects such as nausea, vomiting and dizziness in the group treated with Lavandula in our study and other studies on this drug, it can be concluded that this drug doesnot have serious and common side effects of the opioid analgesics and NSAIDs, and further studies could be used as part of a multimodal analgesic treatment of postoperative pain. Based on findings of our study, it can be concluded that the inhaled Lavender essence may be used as a part of the multimodal analgesic treatment after cesarean section, but it is not recommended using the sole analgesic treatment.
